# Highly Efficient Activation of Peroxymonosulphate by Co and Cu Co-Doped Sawdust Biochar for Ultra-Fast Removal of Bisphenol A

**DOI:** 10.3390/molecules29225296

**Published:** 2024-11-09

**Authors:** Hui Liang, Tongjin Liu, Ruijuan Li, Rumei Li, Yuxiao Zhu, Feng Fang

**Affiliations:** 1 Institute of Plant Protection, Shandong Academy of Agricultural Sciences, Jinan 250100, China; huiliangchem@163.com (H.L.); tongjinliu2024@163.com (T.L.); ruijuanli@163.com (R.L.); rumeili0815@163.com (R.L.); sdgtzyx@163.com (Y.Z.); 2 Shandong Key Laboratory for Green Prevention and Control of Agricultural Pests, Jinan 250100, China

**Keywords:** peroxymonosulphate, ultra-fast degradation, carbon-based catalysts, organic pollutants

## Abstract

The rapid, efficient, and thorough degradation of Bisphenol A (BPA) is challenging. In this study, we prepared an effective peroxymonosulphate (PMS) activation catalyst derived from sawdust containing calcium carbonate. The Co and Cu co-doped sawdust biochar (CoO/CuO@CBC) catalyst could activate PMS quickly, and the degradation rate of BPA reached 99.3% in 5 min, while the rate constant was approximately 30 times higher than in the CBC/PMS and CoCuO_x_/PMS systems. Moreover, the interaction between CoO, CuO, and CBC endows the CoO/CuO@CBC catalyst with excellent catalytic performance under different conditions, such as initial pH, temperature, water matrix, inorganic anions, and humic acid, which maintained fast PMS activation via the cyclic transformation of Cu and Co for BPA degradation. The results demonstrated that both the radical (•O_2_^−^ and •SO_4_^−^) and non-radical (^1^O_2_) pathways participate in the degradation of BPA in the CoO/CuO@CBC/PMS system. The efficient and stable degradation over a wide range of pH, temperature, and aqueous matrices indicates the potential application of the CoO/CuO@CBC catalyst.

## 1. Introduction

In recent years, the treatment of wastewater containing organic contaminants, which pose significant environmental and health risks, has received increasing attention [1,2,3,4]. Traditional wastewater treatment approaches (such as ultrasonic method [5], biological method [6], and chemical oxidation [7]) have difficulty in removing contaminants like industrial chemical dyes and textile assistants [8]. Bisphenol A (BPA) is an important industrial chemical wildly used in the synthesis of epoxy resins and polycarbonates. However, as a typical endocrine disruptor present in various water sources, BPA is difficult to degrade effectively using conventional wastewater treatment methodologies [9,10]. Therefore, there is an urgent need to develop effective methods for addressing this problem.

Compared with other traditional methods, advanced oxidation processes based on the activation of peroxymonosulphate (PMS) have recently garnered significant interest owing to the mild reaction conditions of PMS as well as its high selectivity and ability to degrade thoroughly [11,12]. It is well known that sulphate radicals (•SO_4_^−^, potential redox 2.5–3.1 V) are more attractive species than hydroxyl radicals (•OH, potential redox 1.8–2.7 V) for the oxidation of organic pollutants [13]. Moreover, other than •SO_4_^−^, •OH, superoxide radicals (•O_2_^−^) and singlet oxygen (^1^O_2_) may also be produced in PMS-mediated catalytic reactions, with the pollutants completely oxidising into CO_2_ and H_2_O [14,15,16].

To date, various activation approaches such as alkalis, carbonaceous materials, ultraviolet irradiation, heat, transition metals, and oxides have been applied to activate PMS [17]. Among these, transition-metal-based catalysts (particularly transition metal oxides) have received considerable attention for removing stubborn organic pollutants owing to their low energy consumption and ease of recycling [18,19]. Recently, Co and Cu oxide catalysts have achieved remarkable results in PMS activation for contaminant degradation. Wang et al. prepared a cage-like Co_3_O_4_-ZnO/C composite with 9 wt.% Co, resulting in 100% BPA removal with a degradation rate of 0.326 min^−1^ in 12 min [20]. It was reported that bamboo leaf-derived porous biocarbon loaded with CoFe_2_O_4_ demonstrated remarkable BPA degradation over a wide pH range and under high-salinity conditions [21]. Yin et al. reported that when CuO nanosheets were incorporated on the surface of scrap steel slag, the obtained catalyst showed an 80.29% removal efficiency for sulphonamide [22]. Wang et al. employed bimetallic Cu-Fe metal organic framework as a catalyst for activating PMS to degrade organic pollutants (phenol, BPA, methyl blue, tetracycline, and sulfamethoxazole), and the removal efficiency was up to 95% in 30 min. This was due to the cooperative effects of Fe and Cu in promoting electron transfer [23]. However, the practical application of these catalysts remains challenging owing to their inefficient electron transfer.

However, owing to their exceptional resistance to acid-base conditions as well as adaptable electronic and physicochemical characteristics, carbonaceous materials have been employed to activate PMS in the form of metal-free catalysis [24,25]. Many reports have shown that composites of metal oxides and carbonaceous materials perform better in PMS activation to remove organic pollutants because the introduction of carbon can reduce the leaching of metal ions, enhance the electron transfer of PMS, and promote its activation [26,27,28]. For instance, graphene layers improve the electron transfer ability of Co_3_O_4_, enhance the stability of the catalyst, and greatly enhance the degradation efficiency of pharmaceuticals when Co_3_O_4_ is encapsulated within graphene sheets, as revealed by Huang et al. [29]. Additionally, a high-performance Co-C catalyst fabricated using a win-win strategy was used to degrade ciprofloxacin (CIP) in a PMS system, and the carbon coating suppressed the leaching of Co^2+^ during catalytic degradation [30]. Although many achievements have been made in recent years, rapid promotion of PMS activation for removing pollutants from water remains a challenge.

In this study, we have adopted a “win-win” strategy to fabricate catalyst using waste sawdust (the sawdust contains calcium carbonate and a few other impurities, which are additives incorporated during the grinding process to enhance the grinding efficiency) as carbon source. CoO and CuO were anchored on calcium-based biochar (CBC) derived from sawdust. The produced CoO/CuO@CBC catalyst exhibited high efficiency for PMS activation, and the BPA removal rate could reach 99.3% in 5 min, which has not been reported before. The catalytic properties of CoO/CuO@CBC are excellent under a range of conditions. Moreover, the primary mechanism of cooperation between radical and non-radical processes for PMS activation in BPA removal was explored. The fabrication of biochar from waste sawdust that induces environmental pressure and the subsequent loading of metal oxides for the degradation of organic pollutants is undoubtedly a highly valuable undertaking, and it presents a new approach for the governance of organic pollutants.

## 2. Results

### 2.1. Characterization of Catalysts

**Figure 1:**
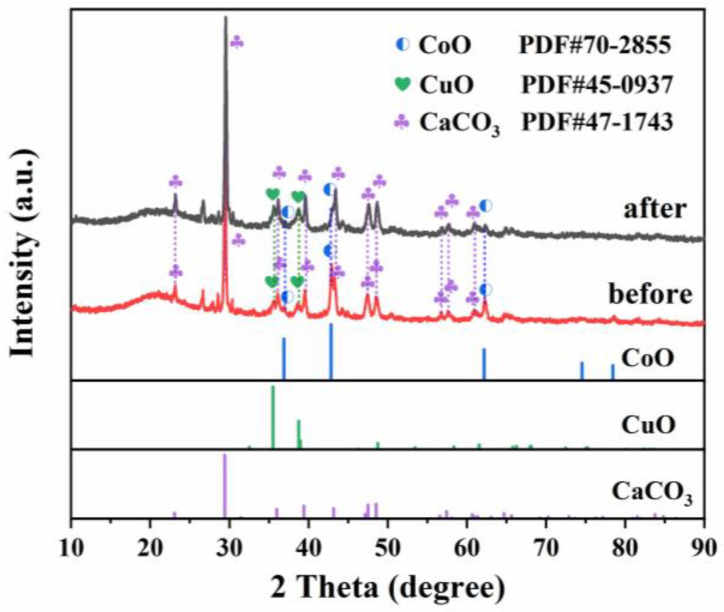
XRD patterns of CoO/CuO@CBC catalysts before and after use.

XRD was used to investigate the crystalline structures of the carbon-based materials. As shown in Figure 1, the main diffraction peaks observed in the XRD spectra of the CoO/CuO@CBC catalysts at 2θ were 23.02°, 29.40°, 35.97°, 39.40°, 43.15°, 47.49°, and 48.51°, which were referred to the (012), (104), (110), (11-3), (202), (018), and (11-6) crystal planes of the standard CaCO_3_ (JCPDS No. 47-1743) [31]. The diffraction peaks positioned at 35.50° and 38.73° correspond to the (002) and (111) crystal planes of CuO (JCPDS No. 45-0937) [32], and the peaks at 36.86°, 42.82°, and 62.17° match well with the (111), (200), and (220) planes of CoO (JCPDS No. 70-2855), respectively. Moreover, the XRD pattern of CoO/CuO@CBC exhibited major diffraction peaks characteristic of CuO and CoO, affirming the retention of the crystalline structures in the synthesised composites.

Compared to the standard reference card, the peak intensities of CuO and CoO markedly decreased in the XRD spectra of the catalysts before and after use, indicating that CaCO_3_ particles were the dominant species and that they might be dispersed in the carbon structures. Furthermore, a hump at approximately 2θ = 21° in the XRD patterns of catalysts before and after use can be indexed to the crystal plane of (002) of poor graphitic carbon as previously reported [33].

The XRD pattern of CBC, CoCuO_x_, CoO@CBC and CuO@CBC is shown in Figure S1. It can be observed that besides the characteristic peaks of calcium carbonate and carbon, there are some impurity peaks in the XRD pattern of CBC, indicating the impurity of the product. It was also observed in the XRD spectra of CoO@CBC and CuO@CBC, which is attributed to the additives in the sawdust. Under the masking of the strong diffraction peaks of impurity and calcium carbonate, the diffraction peaks of CuO and CoO are extremely weak. Moreover, the XRD pattern of CoCuO_x_ obtained through calcination after the combination of cobalt chloride hexahydrate, cupric chloride dihydrate and ammonia, was not matched those of CoO and CuO, suggesting the formation of other cobalt copper oxide.

**Figure 2:**
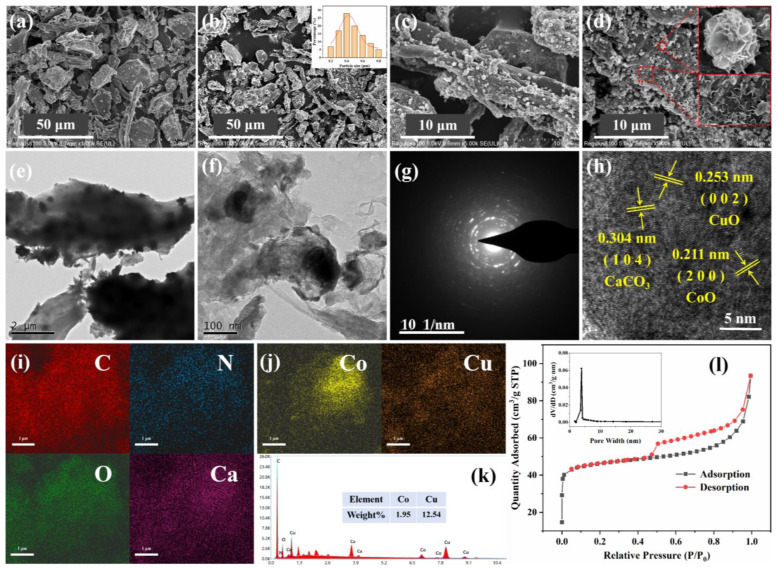
SEM images of (**a**) CBC and (**b**–**d**) CoO/CuO@CBC catalysts (inset: particle size distribution); (**e**,**f**) TEM, (**g**) SAED, (**h**) HRTEM, (**i**,**j**) elemental mapping (C, N, O, Ca, Co, Cu), (**k**) EDS analysis, (**l**) N_2_ adsorption-desorption isothermal curve and pore size distribution curve (inset) of CoO/CuO@CBC catalysts.

As shown in Figure 2, the structural and morphological characteristics of as-synthesised materials were revealed using SEM and HRTEM. The CBC had an irregular morphology and rough surface (Figure 2a, Figure S2a), and some particles retained the tubular wooden structure. From the results of energy dispersive spectroscopy (EDS) analysis of CBC (Figure S2b), it can be seen that apart from C, O and Ca, impurity ions were also contained, resulting in the impurity peaks in the XRD analysis of CBC. After the introduction of Co and Cu, the prepared CoO/CuO@CBC catalyst maintained the morphology of CBC (Figure 2b). In comparison with pure CBC, CuO, and CoO nanoparticles were irregularly attached to the interior and surface of CBC, exhibiting a flower-like or typical wrinkled structure (enlarged in Figure 2c), which is beneficial for exposing more active catalytic sites for the adsorption and degradation of pollutants. The particle size distribution of CoO and CuO ranged from 0.2 to 0.8 micrometres (inset in Figure 2b). Moreover, the specific surface area and pore size distribution were measured using N_2_ adsorption–desorption isotherms, as shown in Figure 2l and Table 1. Based on the adsorption and desorption curves, several hierarchical mesopores with an average diameter of 3.2 nm were found on the CoO/CuO@CBC catalyst, demonstrating a large surface area of 178.1 m^2^/g. In general, the high specific surface area as well as abundant and hierarchical mesopores have a positive effect on the improvement of the catalytic activity [34], which further confirms the SEM results.

The morphologies of CoO/CuO@CBC were further verified by HRTEM, revealing the presence of irregular blocks and flower-like structures (Figure 2e,f), consistent with the SEM results. As shown in Figure 2g, the selected area electron diffraction (SAED) diagram exhibited the polycrystalline nature of the material. CoO/CuO@CBC was confirmed to exist as a composite, wherein the carbon layer was intricately combined with the CoO, CuO, and CaCO_3_ crystal. The crystal structure of CoO/CuO@CBC was revealed in the HRTEM images presented in Figure 2h. In combination with the XRD analysis results, the lattice spacings of 0.211 and 0.253 nm corresponded to the CoO (200) and (002) planes of CuO, respectively, whereas the lattice spacing of 0.304 nm corresponds to the (104) crystal planes of CaCO_3_. Additionally, the SEM-EDS elemental mapping clearly demonstrates that C, N, O, Ca, Co, and Cu are uniformly distributed throughout the catalyst (Figure 2i,j), further proving the successful doping of Co and Cu on the CBC. Furthermore, the energy spectrum of CoO/CuO@CBC indicated that the contents of Co and Cu were approximately 2% and 12.5%, respectively, and this was obviously different from the amounts added during synthesis, which was caused by the incomplete loading of metal ions during the catalyst preparation process.

**Figure 3:**
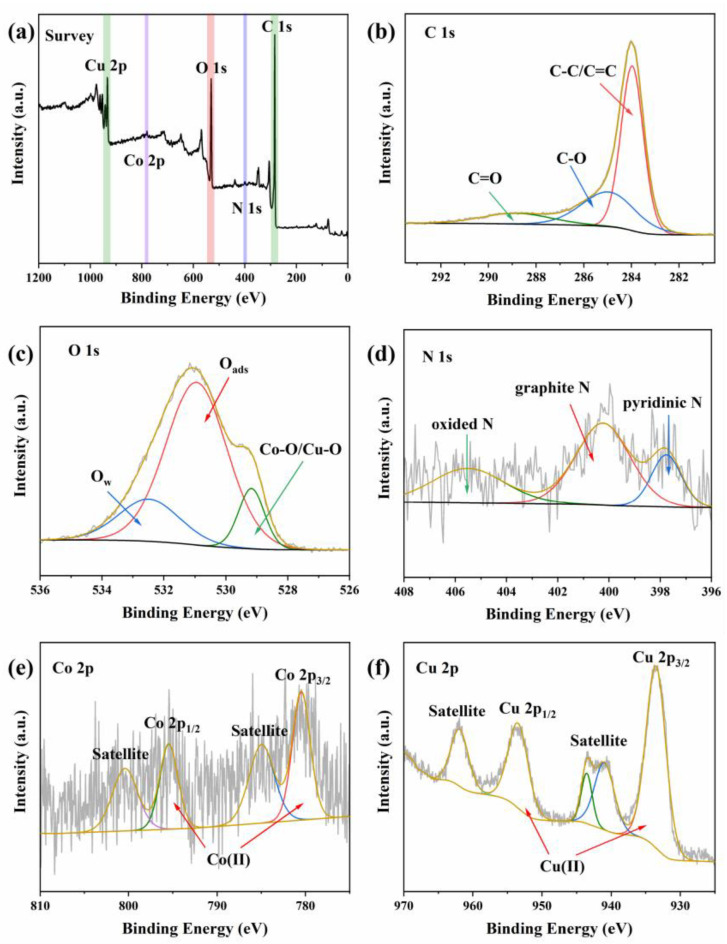
XPS patterns of CoO/CuO@CBC catalysts before use: (**a**) survey spectra, (**b**) C 1s, (**c**) O 1s, (**d**) N 1s, (**e**) Co 2p, and (**f**) Cu 2p.

An XPS test was introduced for comparison of elemental composition and valence band structure of CoO/CuO@CBC before use (Figure 3). As depicted in Figure 3a, strong signals of C 1s, N 1s, O 1s, Co 2p, and Cu 2p were revealed in survey spectra, indicating the basic chemical composition of CoO/CuO@CBC. Additionally, impurity peaks of other elements can also be observed in the spectrum, which is consistent with the results of XRD and EDS analysis. Figure 3b shows high-resolution XPS spectra of C 1s and three peaks at 283.73, 284.98, and 288.88 eV denoted by C-C/C=C, C-O, and C=O, respectively [33]. In the O 1s envelop, three peaks at 529.18, 530.97, and 532.53 eV were attributed to lattice oxygen (Co-O/Cu-O), adsorbed oxygen (O_ads_), and adsorbed water molecules (O_w_) [35,36]. Furthermore, the N 1s spectra of CoO/CuO@CBC comprised three peaks with binding energies of 397.72, 400.26, and 405.51 eV, which belonged to pyridinic N, graphitic N, and oxidized N, respectively [37]. Notably, graphitic N in the catalyst is beneficial for the activation of PMS in organic pollutant degradation, possibly because graphitic N can break the inertness of the graphitic carbon matrix and accelerate electron transfer among adjacent carbon atoms [38]. As presented in Figure 3e, two main peaks of Co 2p_1/2_ and Co 2p_3/2_ were observed at 780.48 and 795.45 eV, respectively, which were ascribed to Co(Ⅱ). A further two satellite peaks at 784.97 and 800.40 eV were also observed [39,40]. Figure 3f shows the XPS spectra of Cu 2p, with the characteristic peaks at 933.52 and 953.45 eV attributed to Cu(Ⅱ), and the peaks at 940.98, 943.53, and 961.88 eV considered satellite peaks of Cu(Ⅱ) oxide species, suggesting the existence of CuO in the catalyst [41,42,43]. In general, the XPS results further confirm the XRD results which indicated the presence of CoO and CuO nanoparticles in CoO/CuO@CBC.

### 2.2. Catalytic Performance

**Figure 4:**
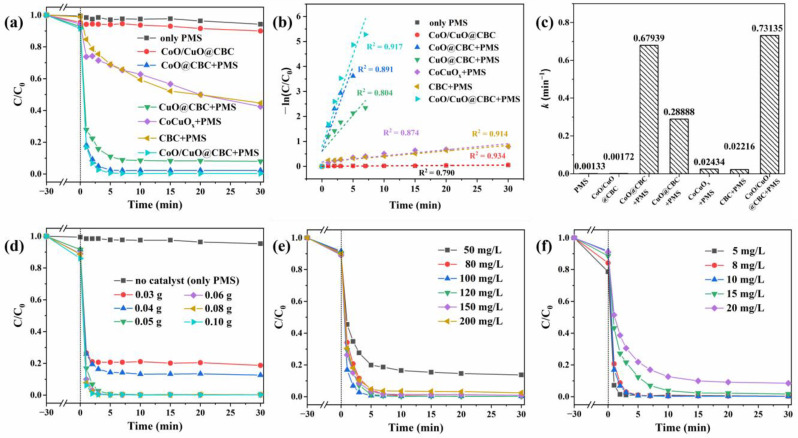
(**a**) Degradation efficiencies, (**b**) degradation kinetic curves, and (**c**) the apparent rate constants k of BPA degradation in the different systems. The effects of (**d**) catalyst dosage, (**e**) PMS concentration, and (**f**) initial BPA concentration on BPA degradation. Reaction conditions: Catalyst = 0.05 g, BPA = 10 mg/L, PMS = 100 mg/L, initial pH = 7.2 (unadjusted), T = 25 °C.

The catalytic performance of the different systems was evaluated for the degradation of BPA. As exhibited in Figure 4a, the degradation efficiencies were 5.76% and 10.01% after 30 min when the system contained only PMS or CoO/CuO@CBC catalysts, respectively, indicating that effective active species could not be generated for BPA oxidization [44]. In addition, the degradation efficiency of 10.01% also indicates that adsorption of the CoO/CuO@CBC catalyst played a minor role in BPA removal. In contrast, the removal of BPA was significantly improved in the presence of both PMS and the catalyst. Compared to CoCuO_x_ (57.57%) and CBC (55.32%), it was shown that both CoO@CBC (97.76%) and CuO@CBC (91.98%) exhibited much better catalytic performance in the PMS system. Furthermore, the degradation efficiency of BPA reached 99.30% within 5 min (total removal of 99.72%) in the CoO/CuO@CBC/PMS system, which displayed the best catalytic performance. This was probably because the special structure of the CoO/CuO@CBC catalyst could expose more active sites and more effectively improves electron transfer. As dedicated in Figure S3, the BPA absorption peak gradually decreased with continued reaction time; meanwhile, the maximum absorption peak had no shift during degradation. The reaction rate constants (k) were derived using a pseudo-first-order kinetic model and are presented in Figure 4b,c. The kinetic constant k of the CoO/CuO@CBC/PMS system was the highest, and was 33, 30, 2.5, and 1.1 times that of the CBC/PMS, CoCuO_x_/PMS, CuO@CBC/PMS, and CoO@CBC/PMS systems, respectively. This revealed that the interaction between CoO, CuO, and CBC was highly beneficial for activating PMS for BPA degradation.

The effects of the catalyst as well as PMS and BPA concentrations on BPA degradation are depicted in Figure 4d–f. The BPA degradation efficiency and the apparent rate constants k increased with increasing catalyst contents (Figure 4d and Figure S4a). Furthermore, when the concentration of PMS increased from 50 to 200 mg/L, the removal rate of BPA initially increased and then decreased (Figure 4e). This phenomenon can be attributed to the saturation of the active sites and self-scavenging of excess radicals, thereby inhibiting the BPA degradation process at high PMS concentrations [45]. At the same time, the catalytic activity of CoO/CuO@CBC and the value of k decreased significantly with increasing BPA concentrations, as shown in Figure 4f and Figure S4c. Considering both degradation effectiveness and economic efficiency, a catalyst dosage of 0.05 g, PMS concentration of 100 mg/L, and BPA concentration of 10 mg/L were selected for further experiments.

### 2.3. Analysis of Environmental Impact Factors

**Figure 5:**
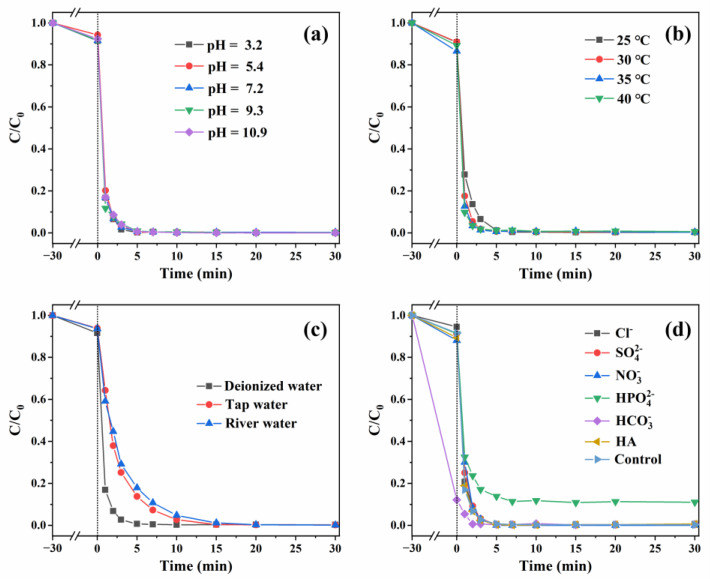
Effects of (**a**) initial pH, (**b**) temperature, (**c**) water matrix, and (**d**) inorganic anions and humic acid on BPA degradation in the CoO/CuO@CBC/PMS system. Reaction conditions: catalyst = 0.05 g, BPA = 10 mg/L, PMS = 100 mg/L, initial pH = 7.2 (unadjusted), inorganic anions = 20 mM, HA = 10 mg/L, T = 25 °C.

The pH level is an essential factor that must be considered in the degradation of organic pollutants. Figure 5a shows the influence of the initial pH level on the BPA degradation performance. It can be seen that there is no notable change in the efficiency of BPA removal at pH values ranging from 3.2 to 10.9, which exhibited certain advantages in actual wastewater treatment and environmental protection. Nevertheless, the reaction rate constant k was gradually decreased with increasing pH values (Figure S5a). As per previous reports, pollutant degradation could be suppressed at acidic conditions, attributed to excessive H^+^ ions having a scavenging effect on •OH and •SO_4_^−^ [46,47,48]. Besides, some studies have shown that in the presence of excess OH^−^ at high pH values, •SO_4_^−^ could convert to it to •OH; •OH has a relatively short lifetime, thus it inhibits the removal efficiency of pollutants [49,50]. However, it is worth noting that the degradation efficiency of BPA reached 99.8% and 99.3% in just 5 min in this study at pH 3.2 and 10.9, and the corresponding reaction constant k was 1.19869 and 0.49676 min^−1^, respectively. The catalytic performance of CoO/CuO@CBC catalyst is excellent under both acidic and alkaline conditions, and it is conspicuously superior to that reported in the previous literature (as shown in Table 2). The possible reasons could be the following: on one hand, a large amount of •OH was generated in the CoO/CuO@CBC/PMS system but did not participate in the oxidative degradation of BPA; on the other hand, although •SO_4_^−^ participates in the degradation process of BPA, •SO_4_^−^ is not the primary active species, which was confirmed from the capture agent experiment. Generally, the reaction rate of the catalytic process increases with the increasing reaction temperature because the reaction temperature not only improves the PMS activation efficiency, but also reduces the reaction barrier, accelerating the decomposition of pollutants [51,52]. As illustrated in Figure 5b, an elevated reaction temperature only slightly improves the degradation efficiency, as well as the reaction rate constant also insignificant changed (Figure S5b), reflecting the excellent ability of the CoO/CuO@CBC catalyst.

To evaluate the practical application potential of CoO/CuO@CBC for the degradation of BPA, CoO/CuO@CBC was used to degrade BPA in different water matrices (Figure 5c). Three types of water matrices were selected: deionised water (DW), tap water (TW), and river water (RW). Compared to DW as a water matrix, the degradation of BPA in TW and RW was slightly inhibited, which might be due to the effect of inorganic anions and humic acid (HA) on TW and RW, resulting in the obvious decreased degradation rate (Figure S5c). However, the removal efficiency of BPA still reached 99.7% within 20 min in the two water matrices as well as in DW.

In practical applications, anions and organic matter in wastewater can affect the degradation of organic pollutants and the activation of persulfate. Therefore, it was necessary to investigate the influence of Cl^−^, SO_4_^2−^, NO_3_^−^, HPO_4_^2−^, HCO_3_^−^, and HA on the catalytic degradation of BPA in the CoO/CuO@CBC/PMS system, and the results are shown in Figure 5d. Cl^−^, SO_4_^2−^, NO_3_^−^, HCO_3_^−^, and HA showed negligible influence on BPA degradation, whereas HPO_4_^2−^ significantly reduced the degradation efficiency. This is because phosphate could be absorbed on the surface of CoO/CuO@CBC instead of PMS [53,54] and it could also form complexes with metal ions, which can then cover the active sites on the catalyst surface, thereby hindering the removal of pollutants [55,56]. As shown in Figure S5d, the effect of the anions and HA on the degradation rate of BPA was highly significant.

**Table 2:** Comparison between CoO/CuO@CBC and the previously reported catalysts in the catalytic performance.

Catalyst	Organic Polltants	Optimal Reaction Conditions	pH	Degradation Efficiency	The Apparent Rate Constant K (min^−1^)	Stability	Reference
NiFe_2_O_4_	2,4-dichlorophenox-yacctic acid (2,4-D)	2,4-D = 0.5 mg/L catalyst = 100 mg/L oxidant = 240 mg/L	3.1	3.1%, 15 min	0.0023	80.70%, 3 recycles	[57]
			7.2	97.5%, 15 min	0.2254		
			10.0	42.4%, 15 min	0.0292		
Cu_SA_-N-C	sulfamethoxazole (SMX)	SMX = 10 μM catalyst = 0.2 g/L oxidant = 0.52 mM	3	about 60%, 30 min	0.026	/	[58]
			7	100%, 20 min	0.21		
			10	about 50%, 30 min	0.018		
Mn/C-KOH	phenol	phenol = 30 mg/L catalyst = 300 mg/L oxidant = 200 mg/L	11	82, 50 min	/	74%, 5 recycles	[59]
			7	95%, 50 min	0.209		
Activated biochars-800	chloramphenicol (CAP)	CAP = 0.31 mM catalyst = 2.0 g/L oxidant = 6.2 mM	2.0	100%, 150 min	0.02477	/	[60]
			9.0	90%, 200 min	0.01504		
ZnO-Co_3_O_4_/C	BPA	BPA = 20 mg/L catalyst = 0.2 g/L oxidant = 400 mg/L	/	100%, 12 min	0.326	86%, 3 recycles	[20]
CoFe_2_O_4_@PBC_X_	BPA	BPA = 20 mg/L catalyst = 200 g/L oxidant = 200 mg/L	11	100%, 9 min	0.23219	59.18%, 5 recycles	[21]
CoO-C-500	CIP	CIP = 10 mg/L catalyst = 50 mg/L oxidant = 0.5 mM	3	near 100%, 30 min	0.20237	near 95%, 4 recycles	[30]
			7	100%, 30 min	0.25382		
			11	near 100%, 30 min	0.19026		
CoO/CuO@CBC	BPA	BPA = 10 mg/L catalyst = 0.833 g/L oxidant = 100 mg/L	3.2	99.8%, 5 min	1.1987	97.7%, 5 recycles	this study
			7.2	99.7%, 5 min	0.7314		
			10.9	99.3%, 5 min	0.4968		

### 2.4. Universality and Stability of Catalyst

**Figure 6:**
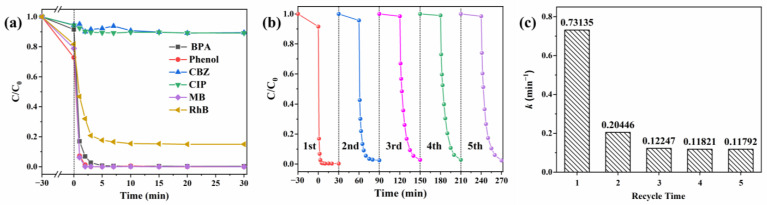
(**a**) Degradation performance of CoO/CuO@CBC catalyst on different organic pollutants, (**b**) recycling experiments, and (**c**) the apparent rate constants k of BPA degradation in the recycling experiments. Reaction conditions: catalyst = 0.05 g, BPA = 10 mg/L, phenol = 10 mg/L, CBZ = 10 mg/L, CIP = 10 mg/L, MB = 50 mg/L, RhB = 50 mg/L, PMS = 100 mg/L, initial pH = 7.2 (unadjusted), T = 25 °C.

To verify the catalytic ability of the CoO/CuO@CBC catalyst, we further evaluated the universality of the CoO/CuO@CBC/PMS system on the degradation of other pollutants, such as phenol, CBZ, CIP, MB, and RhB. From Figure 6a, it can be seen that there was no significant effect on the degradation of CBZ and CIP by the CoO/CuO@CBC/PMS system. However, CoO/CuO@CBC exhibited good PMS activation to remove phenol and MB (removal efficiency: phenol 100%, MB 100%), whereas it demonstrated a satisfactory removal effect on RhB (removal efficiency 85%).

Recoverability is one of the important factors affecting the practical use of catalysts, and the stability of the CoO/CuO@CBC catalyst was investigated after recovery. The XRD pattern of the CoO/CuO@CBC catalyst is illustrated in Figure 1. Compared to the fresh catalyst, the peak position and strength were not significantly affected. As shown in Figure 6b, the final removal efficiency of BPA decreased slightly from 99.7% to 97.7% after five cycles, while the k values of the CoO/CuO@CBC catalyst decreased from 0.73135 min^−1^ to 0.11792 min^−1^ (Figure 6c), indicating the decreased activation ability of the CoO/CuO@CBC catalyst for PMS. This may have occurred due to the leaching of copper and cobalt ions in the catalyst during the reaction process [61], which was verified in the EDS analysis in Figure S6. Furthermore, compared with those of the reported high-performance catalytic materials (Table 2), CoO/CuO@CBC showed outstanding catalytic performance. According to the above results, it is worth believing that the CoO/CuO@CBC catalyst has good stability and practical application prospects.

### 2.5. Catalysis Mechanisms

**Figure 7:**
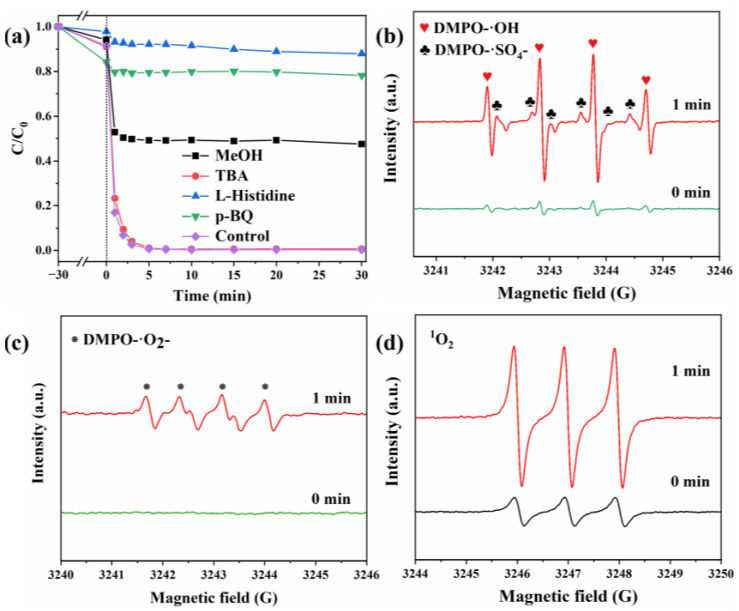
(**a**) Experiment studying the trapping of active species during BPA oxidation on CoO/CuO@CBC (Reaction conditions: Catalyst = 0.05 g, BPA = 10 mg/L, PMS = 100 mg/L, initial pH = 7.2 (unadjusted), p-BQ = 2 mM, L-Histidine = 2 mM, MeOH = 25 mM, TBA = 25 mM, T = 25 °C). ESR spectra of (**b**) DMPO-•OH and DMPO-•SO_4_^−^, (**c**) DMPO-•O_2_^−^, and (**d**) TEMP-^1^O_2_.

The reactive oxygen species involved in the CoO/CuO@CBC/PMS system and their contribution to BPA degradation are further illustrated in Figure 7. Catalytic activity measurement of the CoO/CuO@CBC was carried out in the presence of various radical scavengers, with methanol employed to quench •SO_4_^−^ and •OH, whereas TBA, L-histidine, and p-BQ were used to quench •OH, ^1^O_2_, and •O_2_^−^, respectively [62,63]. As shown in Figure 7a, the BPA removal efficiency decreased from 99.3% to 50.8%, 7.3% and 20.6% after 5 min in the presence of MeOH, L-histidine, and p-BQ, respectively. However, the addition of 25 mM TBA showed a negligible influence on BPA oxidation, indicating that •OH was not the primary active species in the CoO/CuO @CBC-mediated system. The difference between TBA and MeOH provided a preliminary verification for the existence of •SO_4_^−^ free radicals, which was integrally responsible for BPA degradation in the reaction system. Furthermore, L-histidine and p-BQ showed an obvious inhibitory effect, highlighting the predominant roles of ^1^O_2_ and •O_2_^−^ for BPA degradation within the CoO/CuO@CBC/PMS system.

In order to further verify the type of the active substance in the system, ESR measurement was conducted using DMPO and TEMP as trapping agents. EPR signals of DMPO-•SO_4_^−^, DMPO-•O_2_^−^, and TEMP-^1^O_2_ were consistently observed in the reaction system (Figure 7b–d), confirming that •SO_4_^−^, •O_2_^−^, and ^1^O_2_ indeed participated in the BPA degradation process to varying degrees. Moreover, typical peaks with an intensity ratio of 1:2:2:1 assigned to DMPO-•OH were detected in the CoO/CuO@CBC/PMS system, indicating the presence of •OH. Based on this finding in combination with the results shown in Figure 7, it is suggested that the contribution of various radicals to BPA degradation increased in the order of ^1^O_2_ > •O_2_^−^ > •SO_4_^−^ > •OH.

Co(Ⅱ) + HSO_5_^−^ → Co(Ⅲ) + •SO_4_^−^ + OH^−^ (1)

Cu(Ⅱ) + HSO_5_^−^ → Cu(Ⅰ) + •SO_5_^−^ + H^+^ (2)

Co(Ⅲ) + HSO_5_^−^ → Co(Ⅱ) + •SO_5_^−^ + H^+^ (3)

Cu(Ⅰ) + HSO_5_^−^ → Cu(Ⅱ) + •SO_4_^−^ + OH^−^ (4)

Cu(Ⅰ) + HSO_5_^−^ → Cu(Ⅱ) + SO_4_^2−^ + •OH (5)

2•SO_5_^−^ → 2SO_4_^2−^ + ^1^O_2_ (6)

Cu(Ⅰ) + O_2_ → Cu(Ⅱ) + •O_2_^−^ (7)

Co(Ⅱ) + O_2_ → Co(Ⅲ) + •O_2_^−^ (8)

•SO_4_^−^ + ^1^O_2_ + •O_2_^−^ + BPA → small molecules → CO_2_ + H_2_O (9)

**Figure 8:**
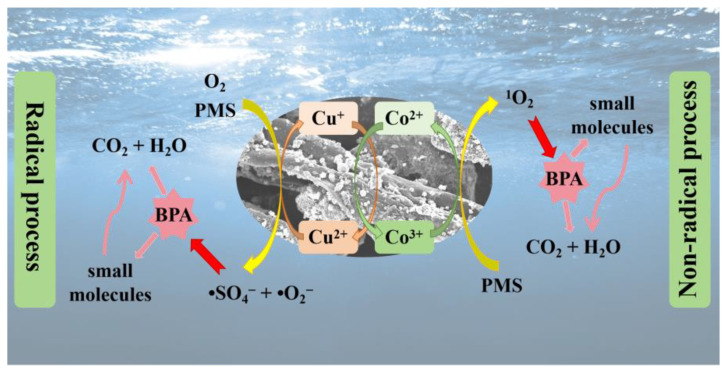
Possible mechanism for activation of PMS by CoO/CuO@CBC.

Based on the trapping experiment results, the catalytic mechanism of CoO/CuO@CBC activation of PMS for BPA degradation is proposed in Figure 8. Radical and non-radical processes for BPA degradation were also performed. First, Co(Ⅱ) on the surface of the CoO/CuO@CBC catalyst acts as the catalytic centre for the activation of PMS to produce •SO_4_^−^, coupled with Co(Ⅲ) formation via electron transfer (Equation (1)) [64,65]. Cu(II) and Co(III) directly reacted with PMS to form Cu(I) and Co(II) (Equations (2) and (3)) [66], respectively, with the newly generated Cu(II) further participating in the activation of PMS (Equations (4) and (5)) [41,67]. This maintained fast PMS activation by the cyclic transformation of Cu and Co for BPA degradation. This process led to the production of Co(II), Co(III), Cu(I), and Cu(II), as shown in the XPS spectrum of the catalyst after use (Figure S5). Subsequently, the produced •SO_5_^−^ in turn generated ^1^O_2_ as shown in Equation (6) [65]. Furthermore, self-decomposition of PMS, enhanced by CoO/CuO@CBC, could contribute to ^1^O_2_ formation [64]. Previous work showed that dissolved oxygen could react with Cu(Ⅰ)/Co(Ⅱ) to form •O_2_^−^ (Equations (7) and (8)) [67,68]. Because of its high specific surface area, hierarchical mesopores, and excellent conductivity, CBC can not only afford more adsorption and activation sites for PMS and BPA but also serve as channels for electron transfer during the oxidation process. Eventually, the reactive species such as •SO_4_^−^, ^1^O_2_, and •O_2_^−^ generated in these processes attract BPA, forming various small molecule intermediates, which are further oxidised into CO_2_ and H_2_O (Equation (9)). In conclusion, the synergistic interaction between CBC and metals leads to the effective degradation of BPA in CoO/CuO@CBC/PMS system.

## 3. Materials and Methods

### 3.1. Subsection

All the chemicals were used in their original states without additional purification. Detailed information about the materials and chemicals used in this study is provided in Text S1 of the Supporting Information.

### 3.2. Synthesis

#### 3.2.1. Fabrication of CoO/CuO@CBC Composites

First, ultrafine sawdust was washed with deionised water three times and dried in a drying oven at 60 °C for 24 h. Then, 2.5 g of oven dried ultrafine sawdust was added in a 40 mL mixed solution containing 1.19 g of cobalt chloride hexahydrate and 0.85 g of cupric chloride dihydrate. The mixture was then stirred for 2 h. After filtering, the obtained precipitate was dispersed in 40 mL water, and 2 mL of ammonia solution was slowly added into the reaction system with vigorous stirring for 2 h. Afterwards, the solid green product was filtered and washed with deionised water, followed by drying at 60 °C overnight. Finally, the resulting solids were maintained at an annealing temperature of 700 °C for 3 h in a tubular furnace under nitrogen atmosphere.

#### 3.2.2. Fabrication of CoCuO_x_, CoO@CBC, CuO@CBC, and CBC Catalysts

For comparative analysis, other catalysts were prepared by adjusting the additives (two of the three substances: ultrafine sawdust, cobalt chloride hexahydrate, and cupric chloride dihydrate), labelled CoCuO_x_, CoO@CBC, and CuO@CBC, respectively. The CBC catalyst was prepared by annealing washed ultrafine sawdust in an N_2_ atmosphere.

### 3.3. Characterization

The phase structures of the prepared catalysts were characterised using X-ray diffraction (XRD, Bruker, D8, Bremen, Germany), and their morphologies and microstructures were observed using scanning (SEM, FEI QUANTA 250, Hillsborough, OR, USA) and transmission electron microscopy (TEM; FEI Tecnai G2 F20, Hillsborough, OR, USA), respectively. Element distribution of as-prepared materials was evaluated using attached energy dispersive spectroscopy (EDS, IE450X-Max80, Oxford, UK). X-ray photoelectron spectroscopy (XPS, Thermo Scientific K-Alpha, Waltham, MA, USA) was used to investigate the surface elemental compositions and chemical states. Electron paramagnetic resonance (EPR, Bruker ELEXSYS-580, Bremen, Germany) spectra of the catalysts were obtained to investigate the reactive species generated during the BPA degradation process. The specific surface area and pore size distribution were determined using the N_2_ adsorption–desorption technique (Brunauer–Emmett–Teller (BET), Micromeritics ASAP 2460, Norcross, GA, USA).

### 3.4. Experimental Procedure and Analyses

The catalytic activity of the obtained samples for BPA degradation was determined. The catalytic process was performed as follows: an amount of 0.05 g of the catalyst was added to 60 mL of an aqueous solution of BPA (initial concentration: 10 mg/L) in a glass beaker. The suspension was stirred for 30 min to reach equilibrium. Then, a 1.5 mL PMS solution with a concentration of 4 g/L was added to this system to initiate BPA degradation under continuous stirring. Periodically, 1 mL of the suspension was removed from the system and filtered through 0.45 μm membranes. Subsequently, the degradation was quenched using the same volume of methanol. The residual concentration of BPA was determined using high-performance liquid chromatography (HPLC, Waters e2695) at 278 nm using methanol and water as the mobile phases. The concentration of other pollutants was determined by HPLC and UV-Vis spectrophotometer. Further information concerning the experiments is provided in Table S1 in the Supporting Information. The degradation rate (*η*) of BPA was calculated using the equation *η* = *C*_t_/*C*_0_ × 100%, where *C*_0_ is the initial concentration of BPA before reaction and *C*_t_ is the actual BPA concentration produced at reaction time t. The initial pH adjustments were conducted with 0.1 M HCl and NaOH solutions.

## 4. Conclusions

In this study, the CoO/CuO@CBC catalyst was used for PMS activation to achieve rapid and efficient BPA degradation. Almost complete degradation of 10 mg/L BPA was achieved within 5 min. The catalyst displayed high stability and excellent catalytic performance under different conditions, such as catalyst dosage, PMS concentration, initial BPA concentration, initial pH, temperature, water matrix, inorganic anions, and HA. Furthermore, CoO/CuO@CBC exhibited excellent reusability, with a BPA removal rate of 97.7% after five cycles. A possible reason for this is that cyclic transformation of Cu and Co maintained fast PMS activation for BPA degradation, as demonstrated by the XPS results. In addition, the experiment results confirmed that both radical (•O_2_^−^ and •SO_4_^−^) and non-radical (^1^O_2_) processes participated in the degradation of BPA. This study explored the application of CoO/CuO@CBC in BPA removal and provides new ideas for the design and synthesis of fast and efficient catalysts.

## Figures and Tables

**Table 1:** BET surface area, pore volume, and average pore diameter of CoO/CuO@CBC catalyst.

BET Surface Area (m^2^/g)	Pore Volume (cm^3^/g)	Average Diameter (nm)
178.1	0.09	3.2

## Data Availability

Data are contained within the article and Supplementary Materials.

## Supplementary Materials

The following supporting information can be downloaded at: https://www.mdpi.com/article/10.3390/molecules29225296/s1, Text S1: detailed information about the materials and chemicals used in this study; Table S1: details for analytical methods of pollutants; Figure S1: XRD patterns of CBC, CoCuO_x_, CoO@CBC and CuO@CBC catalysts.; Figure S2: (a) SEM image and (b) EDS analysis of CBC; Figure S3: BPA absorption spectra at various reaction times over the CoO/CuO@CBC catalyst; Figure S4: the apparent rate constants k of BPA degradation in the different conditions: (a) catalyst dosage, (b) PMS concentration, and (c) initial BPA concentration; Figure S5: the apparent rate constants k of BPA degradation in the different conditions: (a) initial pH, (b) temperature, (c) water matrix, and (d) inorganic anions and humic acid; Figure S6: EDS analysis of CoO/CuO@CBC catalysts after used; Figure S7: XPS patterns of CoO/CuO@CBC catalysts after used: (a) Co 2p and (b) Cu 2p.
