# Supplementary material for: Highly Efficient Activation of Peroxymonosulphate by Co and Cu Co-Doped Sawdust Biochar for Ultra-Fast Removal of Bisphenol A
Source: Molecules. 2024 Nov 9;29(22):5296. doi: 10.3390/molecules29225296 (PMC11596690; doi:10.3390/molecules29225296)
Supplement: Supplementary file 1 [file molecules-29-05296-s001.zip › molecules-3277744-supplementary.pdf]

Supplementary Material

Title：Highly efficient activation of peroxymonosulphate by Co and Cu co-doped sawdust biochar for ultra-fast removal of bisphenol A

Text S1: Detailed information about the materials and chemicals used.

Cobalt chloride hexahydrate ($CoCl_2 \cdot 6H_2O$, AR), copric chloride dihydrate ($CuCl_2 \cdot 2H_2O$, AR), ammonia solution (28%), hydrochloric acid (HCl, 37%), humic acid (HA, ≥90%) and potassium peroxymonosulfate (PMS, $KHSO_5 \cdot 0.5KHSO_4 \cdot 0.5K_2SO_4$, ≥47% $KHSO_5$ basis) were provided by Macklin Biochemical Technology Co., Ltd. Bisphenol A (BPA, >99%), phenol, ciprofloxacin (CIP, >98%), carbamazepine (CBZ, >98%), methylene blue (MB, AR) and rhodamine B (RhB, AR) were obtained from Aladdin Biochemical Technology Co., Ltd.. The ultrafine wood powder (contain $CaCO_3$) was purchased on line. The other analytical grade reagents such as sodium hydroxide (NaOH), sodium nitrate ($NaNO_3$), sodium sulfate (Na_2SO_4), sodium phosphate dibasic (Na_2HPO_4), sodium bicarbonate ($NaHCO_3$), sodium chloride (NaCl), tert-butyl alcohol (TBA), methanol (MeOH), p-benzoquinone (p-BQ) and L-histidine were purchased from Sinopharm Chemical Reagent Co., Ltd. All the chemicals were used without a further treatment. The deionized water produced by Taiping-M water system was used throughout the experiments.

Table S1. Details for analytical methods of pollutants**.**

Pollutantants/Analysis method	Mobile phase (V%:V%)	UV detector (nm)	Flow rate (mL/min)
BPA/HPLC	water : methanol = 30:70	278	1.0
Phenol/HPLC	water : methanol = 25:75	254	1.0
CBZ/HPLC	water : methanol = 20:80	283	1.0
CIP/HPLC	water : acetonitrile = 80:20	278	1.0
MB/UV-Vis		663	
RhB/UV-Vis		554	

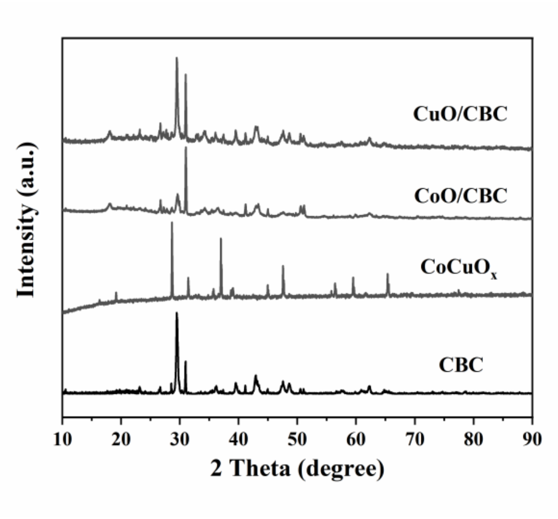

Figure S1. XRD patterns of CBC, CoCuO$_x$, CoO@CBC and CuO@CBC catalysts.

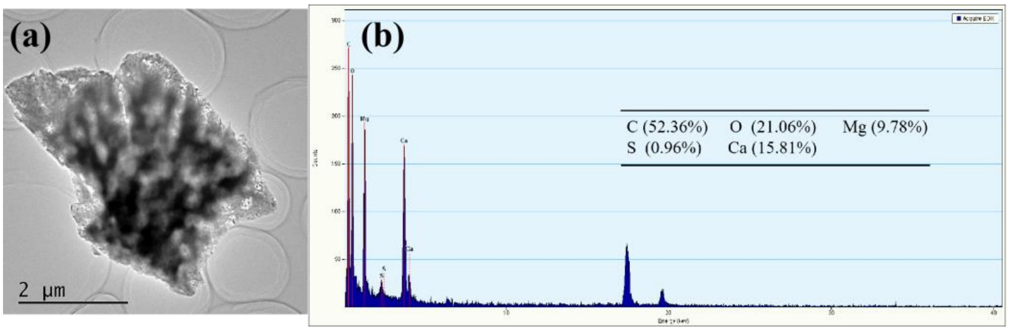

Figure S2. (a) SEM image and (b) EDS analysis of CBC.

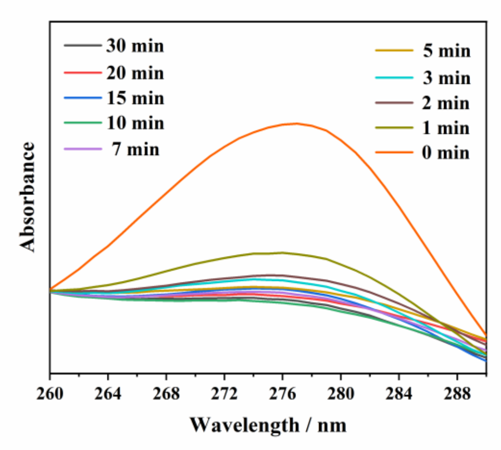

Figure S3. BPA absorption spectra at various reaction times over the CoO/CuO@CBC catalyst.

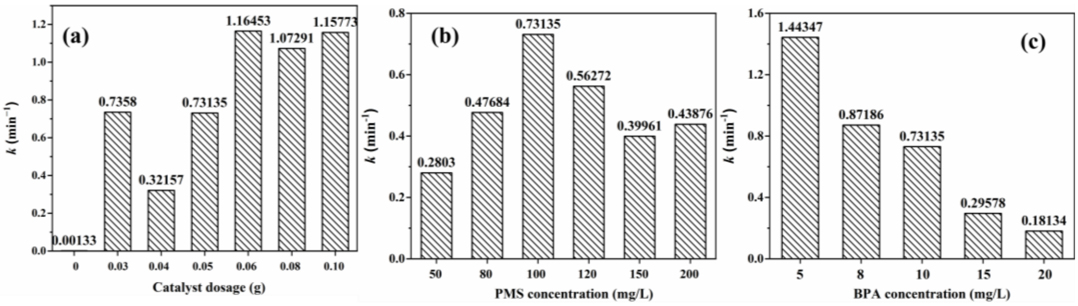

Figure S4. The apparent rate constants k of BPA degradation in the different conditions: (a) catalyst dosage, (b) PMS concentration, and (c) initial BPA concentration.

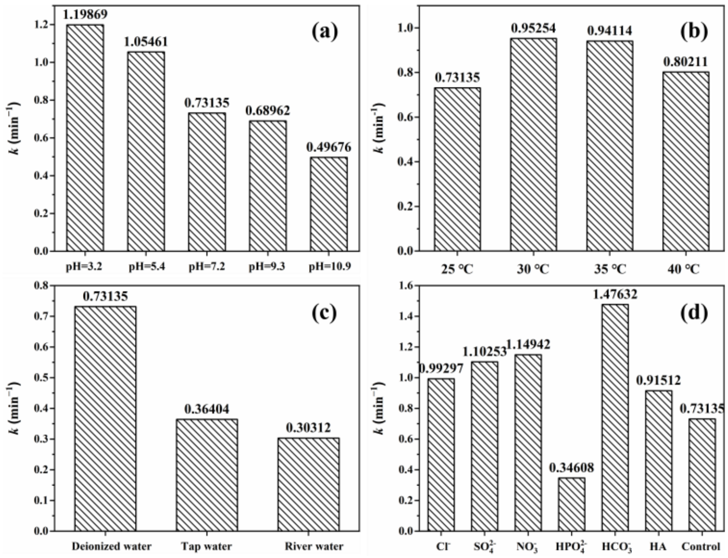

Figure S5. The apparent rate constants k of BPA degradation in the different conditions: (a) initial pH, (b) temperature, (c) water matrix, and (d) inorganic anions and humic acid.

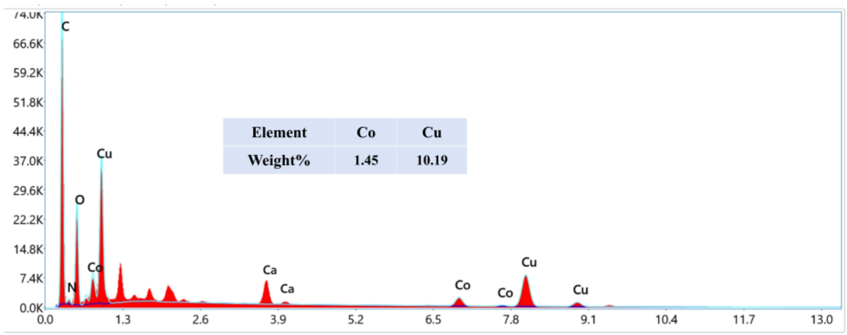

Figure S6. EDS analysis of CoO/CuO@CBC catalysts after used.

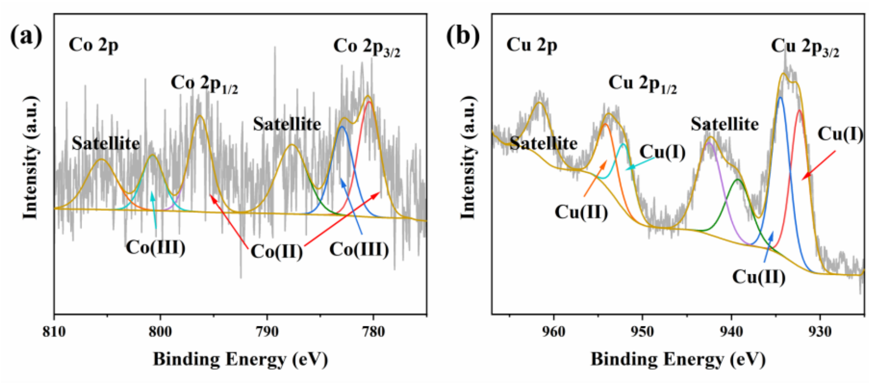

Figure S7. XPS patterns of CoO/CuO@CBC catalysts after used: (a) Co 2p and (b) Cu 2p.
